# Multi-Band Polarization-Insensitive Metamaterial Absorber for Microwave Based on Slotted Structure and Magnetic Rubber

**DOI:** 10.3390/polym14081576

**Published:** 2022-04-13

**Authors:** Kai Li, Haipeng Lu, Mei Bi, Wentao He, Lun Qi, Zhangrong Zhou, Xiaolong Weng

**Affiliations:** National Engineering Research Center of Electromagnetic Radiation Control Materials, School of Electronic Science and Engineering, University of Electronic Science and Technology of China, Chengdu 611731, China; likai_wf@163.com (K.L.); luhaipeng@uestc.edu.cn (H.L.); hewentaowill@163.com (W.H.); lun_qi@126.com (L.Q.); zhouzhangrong2045@163.com (Z.Z.); wengxl59@163.com (X.W.)

**Keywords:** metamaterial absorber (MMA), multi-band, slotted structure, magnetic rubber substrate, impedance matching

## Abstract

A design method of five-band polarization-insensitive metamaterial absorber (MMA) based on the slotted structures and the magnetic rubber is proposed for L-, S-, C-, X-, and Ku-band applications. The slotted structures of the top layer, which evolved from two square rings, are used to excite multi-resonance. The range of the electromagnetic (EM) parameters of a magnetic rubber substrate, which is used to adjust the equivalent impedance of the absorber to match the free space impedance in different bands, is estimated using the impedance matching principle. A series of magnetic rubber substrates based on the estimated EM parameters are prepared and measured, whose thickness is only 0.7 mm, meeting the thin design requirements. The absorption of the proposed absorber greater than 90% at 1.7 GHz, 3.87 GHz, 5.96 GHz, 9.4–10.4 GHz, and 14 GHz is achieved when the doping amount of the carbonyl iron powders is 200%. The absorbing performance of the absorber with measured EM parameter agrees well with the theoretical estimates, which validates the accuracy of the proposed design method.

## 1. Introduction

With the development of electronic and communication technology, the influence of EM radiation and pollution on the environment is increasing [1]. In addition, more and more military equipment is also threatened by EM interference and radar detection. Therefore, the development of EM wave-absorption materials to suppress EM pollution and realize the stealth of the equipment has become a major subject of material science [1]. MMAs break through the traditional design principles and obtain excellent properties by periodic structure design and substrate [2]. In this regard, MMAs have become a research hotspot because of their lightweight, high absorption, wide band, and thinner body [3].

In order to minimize the reflection of incident EM waves, the MMA uses artificially designed periodic units to match the equivalent impedance of free space [4]. The interference theory [5], dielectric loss [6], ohmic loss [7], and other methods are used to improve the absorption performance of the device. Earlier studies in the gigahertz regime are mainly focused on the Salisbury screen [8], Jaumann absorber [9], and Dallenbach absorber [10]. N.I. Landy of Duke University proposed a metal-dielectric-metal that was based on the principle of EM resonance in the year 2008, which is usually referred to as a perfect metamaterial absorber [11]. Following this discovery, research into the development of the EM absorber entered a new phase. Scientists from various countries have conducted extensive research to design lightweight, thin, broadband, and strong MMAs, for example, flexible MMAs [12], broad-angular MMAs [13], broadband MMAs [14,15,16], and multi-band MMAs [17]. To overcome the limit defined by the Rozanov theory [18], lossy absorbers based on the magnetic substrate have been proposed in recent years. For example, L. J. Deng et al. combined the circular frequency selective surface (FSS) with substrate doped with carbonyl iron powder and Co_2_Z to obtain an absorber with absorption peaks in L- and S-band [19]. T. Wang et al. selected the magnetic substrate using the database generated by a genetic algorithm, combined the circular FSS, and obtained the broadband absorption from 2.5 to 17.2 GHz with the 3.1 mm-thick magnetic substrate [20]. N. Gill et al. prepared a 4 mm thick magnetic composite and combined it with different types of resonant patterns to achieve microwave absorption in the range of 2–8 GHz [21]. J. J. Jiang et al. proposed an absorber with resistance-loaded FSS and magnetic substrate, which showed good absorption (>90%) in the wavelength range of 6–18 GHz, with the 2 mm thickness of the magnetic substrate [22]. H. T. Gao et al. designed a metamaterial absorber based on magnetic substrate and resistance rings. Two absorption peaks at 7.58 GHz and 12.75 GHz were achieved, with the magnetic substrate being 2 mm thick [23]. The above studies lay particular emphasis on optimizing the size and shape of the FSS based on a magnetic substrate, so as to obtain a wideband absorption. However, few studies have investigated the influence mechanism and matching method between the EM parameters of the magnetic substrate and the FSS performance.

Considering the factors mentioned above, R. Peymanfar and S. Ghorbanian-Gezaforodi designed the FCBW/β-Co(OH)_2_/PAN nanocomposite and analyzed the microwave-absorbing mechanisms [24]. In this paper, the variation law of EM characteristics of silicone-doped carbonyl iron powder with different proportions is systematically studied. The optimal equivalent EM parameters are obtained through the optimization design of material composition and structure. The EM absorption capacity of the absorber based on the collaborative design of a magnetic rubber substrate and a fractal structure covers five distinct frequency bands. The strong absorption peaks are located at 1.73 GHz, 3.87 GHz, 5.96 GHz, 9.65 GHz, and 14 GHz, respectively, when the doping amount of carbonyl iron powders is 200% (it means that 200 g carbonyl iron powders are doped into 100 g silicone rubber). The thickness (weight) of magnetic materials, which is a main limitation of the application, can be greatly reduced. Our work provides a design method to enhance the impedance matching and significantly improve wideband absorption performance and provides a feasible approach to achieve a polarization-insensitive, thin, and multi-band absorber with potential applications in stealth technology, spectrum identification, and EM compatibility.

## 2. Design and Analysis of the Unit Structure

**Figure 1:**
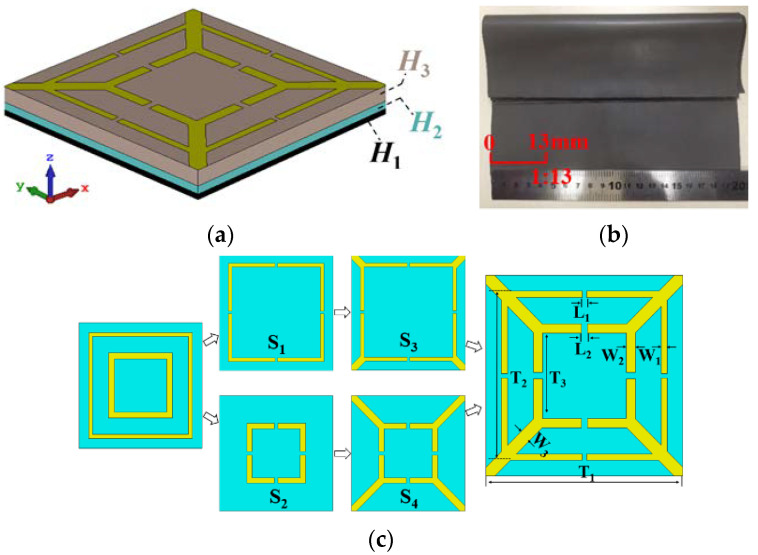
Schematic of the proposed absorber; (**a**) is the side view, (**b**) is the magnetic substrate, and (**c**) is the formation process of the unit cell.

The commercial full-wave EM simulation software with the finite-difference-time-domain (FDTD) solver is used to simulate the proposed MMA with the unit cell boundary in *x*, *y* directions and the open (add space) boundary in *z* direction. As shown in Figure 1a, the absorber is composed of five dielectric layers. The first layer is the slotted structures that are etched on the top side of Fr-4, and Fr-4 is the second layer with *ε*′ = 4.3, tanδε = 0.023, and *H*_3_ = 2 mm. The third, fourth, and fifth layers are made of polymethacrylimide (PMI) foam (*ε*′ = 1.1, *H*_2_ = 1 mm), magnetic substrate (as show in Figure 1b, *H*_1_ = 0.7 mm), and metal backplane (0.017 mm), respectively. Among them, the magnetic substrate is used to control the EM parameters of the absorber, allowing for the equivalent admittance of the absorber to match with the free space. The metal backplane makes the transmission coefficient equal to zero. Figure 1c shows the optimized parameters of the slotted structures, which are *L*_1_ = 0.7 mm, *L*_2_ = 1 mm, *W*_1_ = 1 mm, *W*_2_ = 1.5 mm, *W*_3_ = 2 mm, *T*_1_ = 31.8 mm, *T*_2_ = 26.8 mm, and *T*_3_ = 13.5 mm.

**Figure 2:**
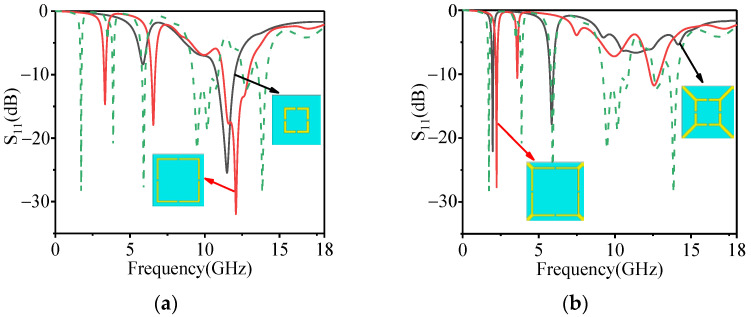
The absorbing effect of each structure acting independently; (**a**) is *S*_1_ and *S*_2_, and (**b**) is *S*_3_ and *S*_4_.

It can be seen from reference [25] that the size, shape, defect, morphology, and medium effect are the vital factors influencing the microwave-absorbing properties. Therefore, in this paper, the opening on the surface of the patch is fully used to excite the resonance, and the superposition of multiple patches is used to expand the number of absorption peaks. The formation process of the slotted structures is shown in Figure 1c. At first, two square metal rings are divided into two notched square metal rings, designated as *S*_1_ and *S*_2_. Then, four corners of *S*_1_ and *S*_2_ are connected with the four corners of the periodic unit cell to form *S*_3_ and *S*_4_, respectively. Finally, *S*_3_ and *S*_4_ are superimposed to form the slotted structures. The solid line in Figure 2a depicts the absorption performance when *S*_1_ and *S*_2_ act alone. The absorption performance of the absorber when *S*_3_ and *S*_4_ act alone is indicated by a solid line in Figure 2b. As can be seen from Figure 2, the absorption peak in L-band is mainly generated by *S*_3_ and *S*_4_. The absorption peak in S-band is mainly generated by *S*_1_. The absorption peak in C-band is mainly generated by *S*_1_ and *S*_4_. The absorption peak in X-band is mainly generated by *S*_1_ and *S*_2_. The absorption peak in Ku-band is mainly generated by *S*_1_ and *S*_3_. As a result, square structures with different sizes can excite resonance at different frequencies, and the slotted design is also helpful to excite resonance, thereby increasing the multi-band of the absorber.

**Figure 3:**
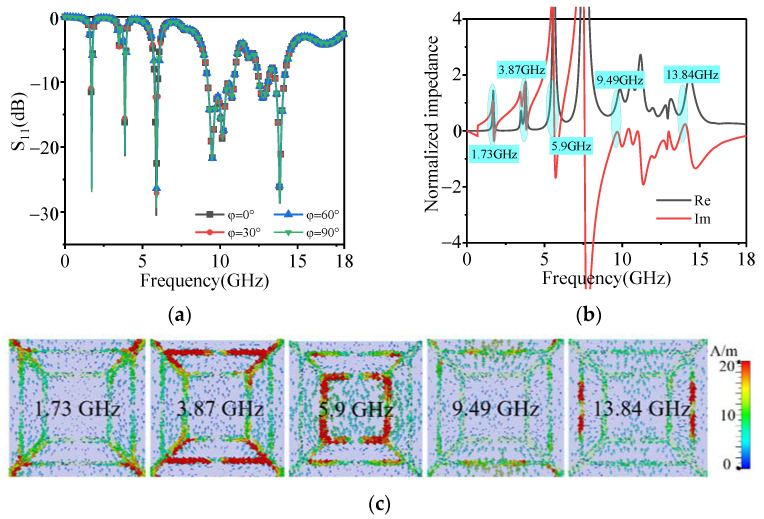
(**a**) S_11_ under different polarization angles, when the incident angle is fixed to 0°. (**b**) The normalized impedance near each resonant point. (**c**) The current at different frequencies in the top surface.

The dotted line in Figure 2 shows the scattering parameters (S_11_), also known as S-parameters, were obtained by theoretical calculation. S-parameters can be used to compute the power coefficients of absorption (*A*), reflection (*R*), and transmission (*T*). *R* = |S_11_|^2^, *T* = |S_21_|^2,^ and *A* = 1−*R*−*T* [26,27]. Due to the metal back at the bottom of the absorber, the EM wave has no transmission, that is, *T* = 0. Therefore, the absorptivity can be expressed as *A*% = 100 (1−*R*) =100 (1−|S_11_|^2^) [26,27]. The absorptivity of the proposed absorber at 1.73 GHz, 3.87 GHz, 5.9 GHz, 9.22–10.89 GHz, and 13.84 GHz is greater than 90% (S_11_ < −10 dB), thus showing multi-band absorption characteristics. Furthermore, the symmetrical design can make the absorber insensitive to the transverse electric and transverse magnetic polarizations. As shown in Figure 3a, the absorption remains unchanged with the variation of polarization angle. This means that the proposed absorber is polarization-insensitive at normal incidence. These characteristics are essential in stealth applications.

The real and imaginary parts of the equivalent impedance of the absorber are shown in Figure 3b. The results revealed that the real part of the equivalent impedance is close to 1, and the imaginary part is approximately 0 in the five resonant frequency point. It indicates that the impedance of the proposed absorber near the five resonance points matches well with the free space. This means that near the five absorption peaks, EM waves can enter the interior of the absorber and lose, which explains the reason for the formation of the five absorption peaks.

To further explain the mechanism of different absorption peaks excited by the slotted structures, the surface current distributions at the five absorption peaks are analyzed (Figure 3c). It can be seen from the figure that the surface current at 1.73 GHz, 9.49 GHz, and 13.84 GHz is mainly distributed in four diagonal metal segments and two slotted square rings, the surface current at 3.87 GHz is mainly distributed in four diagonal metal segments and outer slotted square ring, and the surface current at 5.9 GHz is mainly distributed in the inner slotted square ring. The region with a strong surface current distribution will produce strong EM loss, resulting in strong absorption. The distribution area of the surface current in each band is consistent with the slotted structures corresponding to each band, which further verifies the accuracy of the slotted design.

## 3. Study of EM Parameters Matching Characteristics

**Figure 4:**
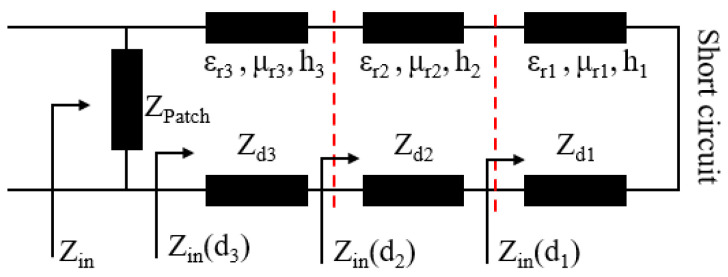
Equivalent circuit of the proposed absorber.

When the equivalent impedance of the absorber matches the free space, the EM wave enters the absorber and is absorbed. The equivalent circuit diagram of the proposed absorber is shown in Figure 4. The bottom metal backplane is equivalent to a short circuit transmission line. The equivalent impedance of the Fr-4 layer, the PMI foam layer, and the magnetic substrate are connected in a series, and then in parallel to the equivalent impedance of the square slotted structures layer. The calculation formula of the input impedance of each layer is given as [28]:

(1) $$Z_{in}\left(d_{n}\right)=Z_{dn}\frac{Z_{in}(d_{n-1})+jZ_{dn}\tan\beta_{n}h_{n}}{Z_{dn}+jZ_{in}(d_{n-1})\tan\beta_{n}h_{n}}(n=1,2,3)$$

Here, $\beta_{n}=\frac{2\pi }{\lambda }\sqrt{\mu_{rn}\varepsilon_{rn}}=\frac{2\pi f}{c}\sqrt{\mu_{rn}\varepsilon_{rn}}(n=1,2,3)$ is the phase shift constant and *c* is the speed of light in free space.

According to Formula (1), the input impedance of magnetic substrate is:

(2) $$Z_{in}\left(d_{1}\right)=jZ_{d1}\tan\beta_{1}h_{1}=jZ_{0}\sqrt{\frac{\mu_{r1}}{\varepsilon_{r1}}}\tan\frac{2\pi fh_{1}\sqrt{\varepsilon_{r1}\mu_{r1}}}{c}$$

The reflection coefficient of the composite absorber is expressed as:

(3) $$RC=\frac{Z_{Patch}\;||\;Z_{in}(d_{3})-Z_{0}}{Z_{Patch}\;||\;Z_{in}(d_{3})+Z_{0}}$$

where *Z_Patch_* is the impedance of the slotted structures and *Z*_0_ = 377 Ω is the characteristic impedance of free space. It can be seen from Formulas (1)–(3) that when the thickness of the magnetic substrate is fixed, the input impedance is a function of EM parameters and frequency. Therefore, the impedance matching and reflection properties of the absorber can be controlled by adjusting the four EM parameters (*ε*′, *ε*″, *μ*′, and *μ*″) of the magnetic substrate.

**Figure 5:**
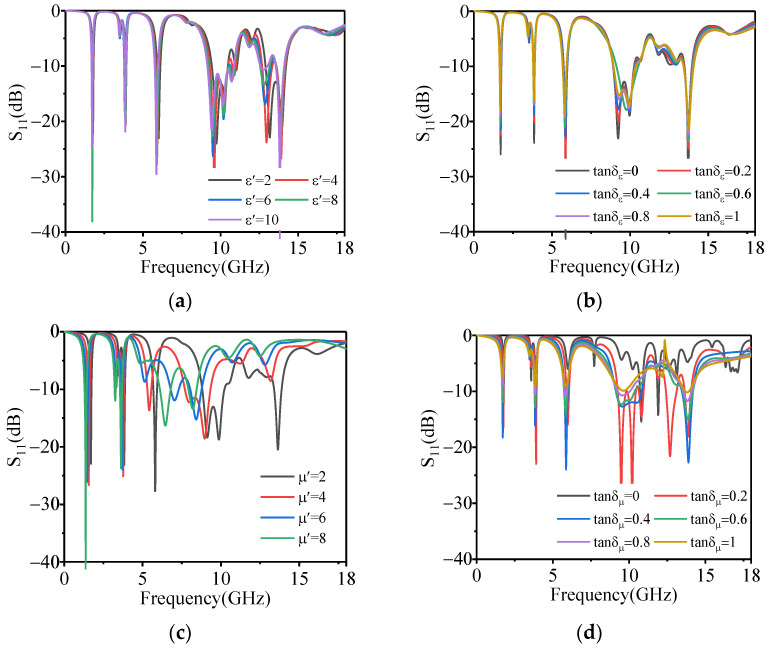
The effect of the EM parameters of the magnetic substrate on the absorption of the proposed absorber; (**a**) is the real part of permittivity, (**b**) is the dielectric loss, (**c**) is the real part of permeability, and (**d**) is the magnetic loss.

**Figure 6:**
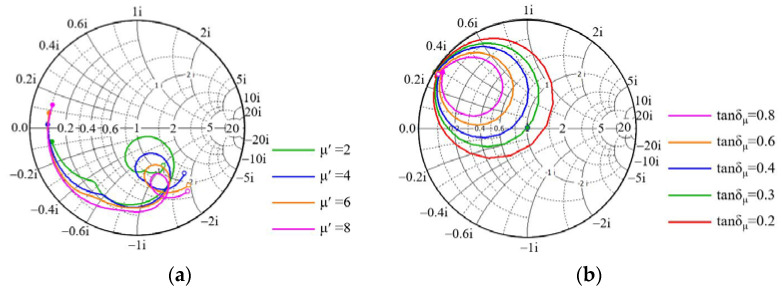
The effect of permeability on matching characteristics of the proposed absorber; (**a**) is the real part in Ku-band and (**b**) is the magnetic loss in L-band.

The EM parameters of the magnetic substrate are optimized to achieve impedance matching in five bands. Referring to the engineering experience of preparing the magnetic substrate, the initial value of the real part of permittivity, the real part of permeability, dielectric loss, and magnetic loss are in the range of 1–10, 1–8, 0–1, and 0–1 respectively. The single variable method is adopted to calculate the S-parameters of the absorber, and the matching between the absorber and free space with different EM parameters is analyzed with the help of a Smith chart. As can be seen from Figure 5a,b, the real part of the permittivity and the dielectric loss has little effect on the microwave absorption. As can be seen from Figure 5c, with the increase of the real part of permeability, the absorption peak moves to a low frequency. This is due to the fact that with the increase of the real part of permeability, the equivalent impedance of the absorber in Ku-band gradually mismatches with free space, which affects the absorption performance (see Figure 6a). Therefore, the real part of permeability that makes the absorption peak in Ku-band less than −10 dB should be kept between 1 and 3. The influence of magnetic loss on the absorption peak of each band is shown in Figure 5d. The absorption peak in five bands first becomes stronger and then weakens with the increase of magnetic loss. The reason for this is that the equivalent impedance of the absorber tends to match first and then gradually mismatch with the increase of magnetic loss, as shown in Figure 6b. Therefore, the magnetic loss that makes the absorption peaks in five bands less than −10 dB should be kept between 0.15 and 0.7.

## 4. Fabrication and Analysis of Magnetic Rubber Substrates

### 4.1. Materials

The main experimental materials were carbonyl iron powder (Fe ≥ 98%, <10 um, JCF1-3) and methyl vinyl silicon rubber (110-2s), provided by Zhuo Innovative Material Co., Ltd., Jilin, China and Dongjue silicone Co., Ltd., Nanjing, China, respectively. Other chemicals included white carbon black, hydroxyl silicone oil, polyvinyl silicone oil, and vulcanizing agent. The white carbon black (>99%, <0.3 um, AEROSIL200) was provided by Degussa AG, Frankfurt, Germany. Hydroxyl silicone oil (Trigonox 101) was purchased from AkzoNobel paint Co., Ltd., Shanghai, China. Polyvinyl silicone oil (XC209) was provided by Xingchi Chemical Co., Ltd., Jinan, China. Vulcanizing agent (DBPMH V110B) was provided by Double bond New Material Co., Ltd., Wuhan, China.

### 4.2. Preparation of Magnetic Rubber Substrates

**Figure 7:**
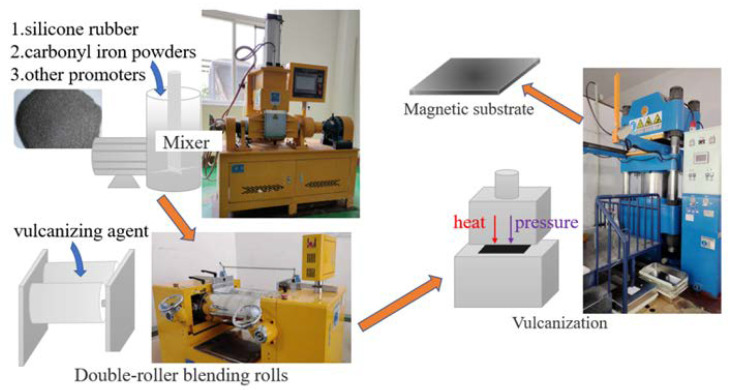
The preparation process of the magnetic substrates.

To prepare the magnetic substrates according to the values of EM parameters mentioned in Section 3 (the real part of permeability should be kept between 1 and 3, and the magnetic loss should be kept between 0.15 and 0.7), a gradient experiment was designed with 100 g silicone rubber as an organic carrier and carbonyl iron powders with different qualities as a magnetic filler. The added amounts of carbonyl iron powder were 50%, 100%, 150%, 200%, 250%, and 300%. The preparation process of the magnetic substrates is shown in Figure 7. Six kinds of magnetic absorbing patches (the thickness is 0.7 mm) with different carbonyl iron powder contents were manufactured through internal mixing, open refining, and vulcanization. Finally, the prepared magnetic absorbing patches were cut into magnetic substrates, with each having the dimensions of 200 × 200 mm^2^.

The preparation of the internal mixing: according to the designed proportion, 100 g silicone rubber, 30 g white carbon black, 1 g vulcanizing agent, 4.5 g hydroxyl silicone oil, 2.5 g polyvinyl silicone oil, and 50 g carbonyl iron powder were successively added to the internal mixer (SY-6212-B-XSM-5L, Shiyan Precision Instrument Co., Ltd., Dongguan, China). They were fully mixed and stirred in the internal mixer to obtain the mixed rubber. Change the addition amount of carbonyl iron powder into 100 g, 150 g, 200 g, 250 g and 300 g in turn, and repeat the above steps to obtain six kinds of rubber compounds with different addition amount of carbonyl iron powder.

The preparation of the open refining: the mixed rubbers were further mixed by a double roll open mixer (SY-6215-Al2-250 × l620, Shiyan Precision Instrument Co., Ltd., Dongguan, China) and rolled into prefabricated blanks. The gap between the two rollers was controlled to be 1.6 mm, and the rotating speed was 10 rpm.

The preparation of the vulcanization: place the prefabricated blanks in the mold of the flat vulcanizer (XJL-P-800T-HS-W1, Xinjinli Machinery Co., Ltd., Shenzhen, China), and set the temperature of the upper mold and the lower mold to 145 °C and 135 °C, respectively. The pressure was set to 100 T, and the vulcanization time was set to 600 s. The size of the final magnetic absorbing material was 350 × 250 × 0.7 mm.

### 4.3. Measurement and Results of the EM Properties

**Figure 8:**
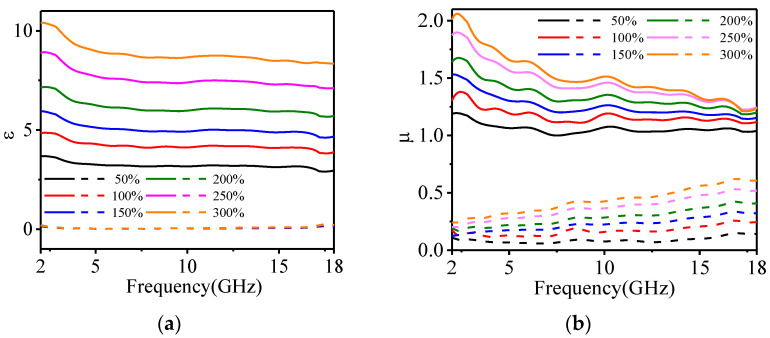
The measured EM parameters of the magnetic substrates; (**a**) is permittivity, and (**b**) is permeability.

It is worthwhile to discuss the complex permittivity (*ε*) and complex permeability (*µ*) in detail for exploring the regulation ability of carbonyl iron powder on EM parameters of magnetic substrate. The free space method [29] is used to test the EM parameters of the magnetic substrate, and the experimental results are shown in Figure 8. The solid line represents the real part of permittivity and permeability, while the dotted line corresponds to the dielectric loss and magnetic loss. Due to the conductivity of carbonyl iron powder, the increasing amount of carbonyl iron powder will increase the propagation of current on its surface, which increases the conduction loss [30,31,32,33]. In addition, the increase of carbonyl iron powder doping will also increase the interfacial polarization, eddy current loss, and resonance loss [30,31,32,33]. Thus, the real part of permittivity and permeability increases as the amount of iron powder increases. Furthermore, the electric loss is not affected by the amount of iron powder, while the magnetic loss increases with the amount of iron powder and frequency.

As the doping amount of carbonyl iron powders increases from 50% to 300%, the average value of the real part of the permittivity increases from 3.21 to 7.57, the average value of the real part of permeability increases from 1 to 1.45, the dielectric loss is maintained at about 0.05, and the average value of magnetic loss increases from 0.087 to 0.37. Therefore, the doping amount of carbonyl iron powder must be greater than 50%. When the amount of carbonyl iron powder doping is 100% to 300%, the impedance matching requirement is satisfied.

**Figure 9:**
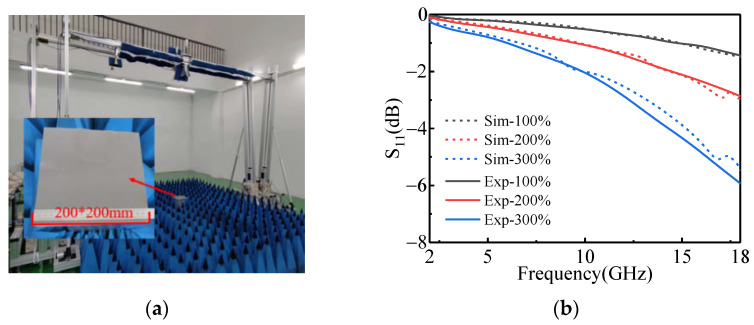
S-parameter analysis of the magnetic substrate; (**a**) is the test scenario and (**b**) is the comparison between S-parameter test results and calculation results.

The S-parameters of the single-layer magnetic substrate samples are measured by arch method [34] with a network analyzer (Keysight N5227B, the test frequency band is 2–18 GHz), as shown in Figure 9a. The measured results are shown by the solid line in Figure 9b. At the same time, the EM parameters of the magnetic substrates obtained from the free space method are introduced into the proposed MMA model to calculate the S-parameters of the single-layer magnetic substrates. The calculation results are shown by the dotted lines in Figure 9b. It can be seen from the figure that with the increase of iron powder doping, the microwave absorption of the single-layer magnetic substrates is improved. Additionally, the measured results are in good agreement with the calculated results. By comparing the S-parameters of the magnetic substrate obtained by the two methods, the accuracy of the EM parameter test and the S-parameter calculation methods are proved.

**Figure 10:**
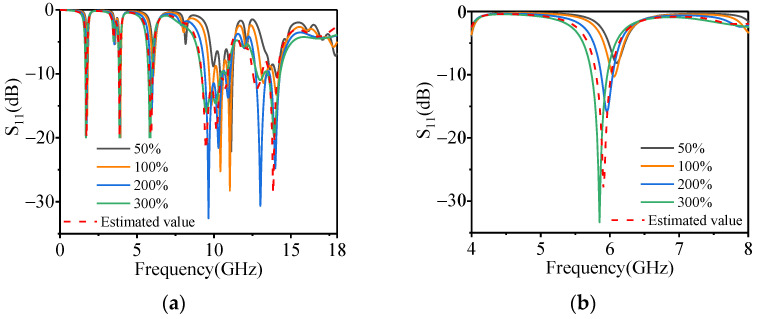
The absorbing properties of the absorber loaded with different types of magnetic substrates; (**a**) is 0–18 GHz, and (**b**) is 4–8 GHz.

Then, the measured EM parameters were introduced into the proposed absorber to calculate its absorbing performance. The calculation results are shown in Figure 10. The S-parameters of the absorber loaded with measured EM parameters are in good agreement with the theoretically estimated values. Regarding the enlarged view of the C-band in Figure 10b, when the doping amount of carbonyl iron powder is 50%, the absorption peak is only −8.1 dB, which means that the absorptivity is less than 90%. As the doping of carbonyl iron powder increases, the absorption peak increases and moves towards lower frequencies. So, when the carbonyl iron powder doping is 100–300%, the absorptivity from L- to Ku-band can be greater than 90%. These findings are in line with the amount of carbonyl iron powder doping determined by impedance matching.

Table 1 and Table 2 compare the proposed absorber with several similar absorbers that have been previously reported. Table 1 compares the absorption bands of the proposed absorber with the multi-band designed based on the multi-resonance structure. As shown in Table 1, only the proposed absorber covers five absorption bands, which take into account both low- and high-frequency bands. Table 2 compares the substrate thickness of the proposed absorber and the magnetic absorber designed based on the magnetic substrate. As shown in Table 2, the proposed absorber significantly reduces the thickness of the magnetic materials without reducing the absorption bands, so it offers a useful combination of multi-band absorption with thin absorption.

### 4.4. The Influence of the Carbonyl Iron Powder Content on Mechanical Properties of Magnetic Substrate

**Figure 11:**
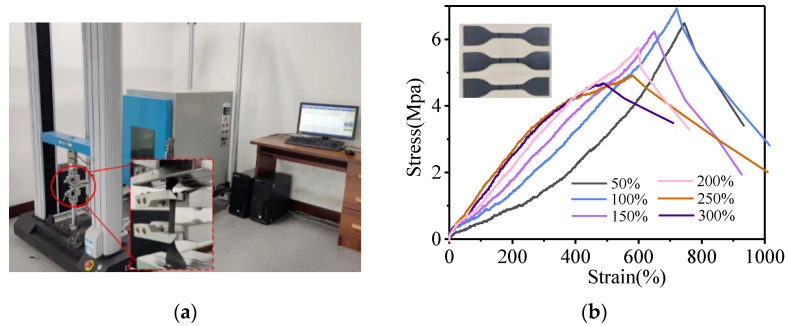
(**a**) A universal strength machine. (**b**) The stress-strain curve.

The mechanical properties of the dielectric/magnetic composites are shown in Table 3, the strength test machine (HD-B607-S, Haida International Instrument Co., Ltd., Dongguan, China) is shown in Figure 11a, and the stress-strain curve is shown in Figure 11b. Before test, the magnetic substrate is cut into the dumbbell shape in Figure 11b. The gauge distance is fixed at 25 mm, and the test speed is 500 mm/min. As can be seen from Figure 11b, the content of the carbonyl iron powder has an effect on the tensile strength, which is mainly reflected in the changes of tensile strength and elongation. When the doping amount of carbonyl iron powder is less than 300%, the tensile strength exceeds 4 MPa and the elongation at break exceeds 400%, which means that the preparation of the MMA based on the magnetic substrate has excellent mechanical properties.

## 5. Conclusions

In conclusion, we propose a five-band, polarization-insensitive, and thin absorber based on magnetic substrate and slotted structures. Multiple absorptions are obtained by designing the slotted structures, and the matching equivalent impedance of the absorber with the free space in different absorption bands by optimizing the EM parameters of the magnetic substrate. The absorption performance of each structure and surface current distribution is used to investigate the working mechanism of the slotted structures. The equivalent transmission-line model and Smith impedance chart are employed to screen magnetic substrates with appropriate EM parameters. To validate the theoretical design, the magnetic substrates with different carbonyl iron powder dopings were prepared, and their EM parameters and S-parameters were measured by free-space method and arch method, respectively. The measured and calculated results show that to achieve more than 90% absorptivity in each absorption band, the doping amount of carbonyl iron powder in the magnetic substrate should be more than 50%. The magnetic substrate has a thickness of only 0.7 mm, which meets the design requirements of a thin and efficient microwave absorber.

## Figures and Tables

**Table 1:** A comparison of the absorption frequency and absorption bands of the proposed absorber with reported multifrequency absorbers.

Ref.	Absorption Frequency (GHz)	Absorption Bands
[35]	5.55, 6.51, 7.71, 9.45, 11.31, 13.11	C-, X-, Ku-
[36]	8.115, 11.4, 15.12	X-, Ku-
[37]	2.538, 7.092, 9.702, 13.302, 15.588	S-, C-, X-, Ku-
Proposed Absorber	1.7, 3.87, 5.96, 9.4–10.4, 14	L-, S-, C-, X-, Ku-

**Table 2:** A comparison of the absorption bands and thickness of the magnetic material of the proposed absorber with reported magnetic absorbers.

Ref.	Absorption Bands	Thickness of Magnetic Material (mm)
[20]	S-, C-, X-, Ku-	3.1
[38]	C-, X-, Ku-, K-, Ka-	2
[39]	S-, C-	3
Proposed Absorber	L-, S-, C-, X-, Ku-	0.7

**Table 3:** The mechanical properties of the magnetic substrates with different contents of carbonyl iron powder.

Doping Amount of Carbonyl Iron Powder (%)	Tensile Strength (Mpa)	Elongation at Break (%)	Elongation at Yield Point (%)
50	6.486	744.51	477.68
100	6.933	720.36	408.86
150	6.238	649.62	309.81
200	5.752	598.4	243.92
250	4.914	580.35	166.75
300	4.676	487.84	165.62

## Data Availability

The data presented in this study are available on request from the corresponding author.
